# Antisense Oligonucleotide Screening to Optimize the Rescue of the Splicing Defect Caused by the Recurrent Deep-Intronic *ABCA4* Variant c.4539+2001G>A in Stargardt Disease

**DOI:** 10.3390/genes10060452

**Published:** 2019-06-14

**Authors:** Alejandro Garanto, Lonneke Duijkers, Tomasz Z. Tomkiewicz, Rob W. J. Collin

**Affiliations:** 1 Department of Human Genetics and Donders Institute for Brain, Cognition and Behaviour, Radboud University Medical Center, 6525GA Nijmegen, The Netherlands; tomasz.tomkiewicz@radboudumc.nl; 2 Department of Human Genetics, Radboud University Medical Center, 6525GA Nijmegen, The Netherlands; lonneke.duijkers@radboudumc.nl

**Keywords:** antisense oligonucleotides, Stargardt disease, inherited retinal diseases, splicing modulation, RNA therapy, ABCA4, iPSC-derived photoreceptor precursor cells

## Abstract

Deep-sequencing of the *ABCA4* locus has revealed that ~10% of autosomal recessive Stargardt disease (STGD1) cases are caused by deep-intronic mutations. One of the most recurrent deep-intronic variants in the Belgian and Dutch STGD1 population is the c.4539+2001G>A mutation. This variant introduces a 345-nt pseudoexon to the *ABCA4* mRNA transcript in a retina-specific manner. Antisense oligonucleotides (AONs) are short sequences of RNA that can modulate splicing. In this work, we designed 26 different AONs to perform a thorough screening to identify the most effective AONs to correct splicing defects associated with c.4539+2001G>A. All AONs were tested in patient-derived induced pluripotent stem cells (iPSCs) that were differentiated to photoreceptor precursor cells (PPCs). AON efficacy was assessed through RNA analysis and was based on correction efficacy, and AONs were grouped and their properties assessed. We (a) identified nine AONs with significant correction efficacies (>50%), (b) confirmed that a single nucleotide mismatch was sufficient to significantly decrease AON efficacy, and (c) found potential correlations between efficacy and some of the parameters analyzed. Overall, our results show that AON-based splicing modulation holds great potential for treating Stargardt disease caused by splicing defects in *ABCA4*.

## 1. Introduction

Stargardt disease (STGD1; MIM:248200) is an autosomal recessive condition affecting the retina, and was first described in 1909 by the German ophthalmologist Karl Stargardt [1]. The clinical hallmark of STGD1 is progressive bilateral impairment of central vision. Impairment in visual acuity and progressive bilateral atrophy of photoreceptors and the retinal pigment epithelium (RPE) are accompanied by the accumulation of toxic fluorescent deposits of lipofuscin in the macula [2,3]. The underlying genetic causes of the disease are mutations in the *ABCA4* gene that encodes the ATP-binding cassette transporter type 4 subfamily A (ABCA4). The *ABCA4* protein belongs to the superfamily of membrane-bound ATP-binding cassette transporters [4]. It translocates the visual cycle metabolites, all-*trans*-retinal and *N*-retinylidene-phosphatidyl ethanolamine (*N*-retinylidene-PE), from the lumen to the cytoplasmic side of photoreceptor disc membranes [5]. The decrease in *ABCA4* activity causes an accumulation of toxic retinal derivatives, which eventually results in RPE and photoreceptor cell death [6,7]. Over 900 disease-associated variants in *ABCA4* have been described [8,9], causing a wide range of phenotypes ranging from STGD1 to cone–rod dystrophy, depending on the severity of the mutation [10,11]. 

STGD1 cases can be explained by biallelic mutations in either the coding sequence or in the intronic regions of *ABCA4* [12]. Around 10% of cases carry intronic variants that result in the insertion of pseudoexons (PEs) into the final *ABCA4* mRNA transcript [4,9,13,14,15,16,17,18,19,20]. Such mutations are an ideal target for antisense oligonucleotide (AON) therapy. AONs are short synthetic RNA molecules that can interfere with the processing of pre-mRNA [21] and thereby modulate splicing. Modified AONs employed to correct splicing defects have been extensively studied in the field of inherited retinal diseases (IRDs) for genes such as *CEP290* [22,23,24,25,26,27]*, USH2A* [28]*, CHM* [29]*, OPN1* [30], or *ABCA4* [18,20,31]. The first splicing modulation strategy described for a retinal disease was targeting a recurring deep-intronic variant (c.2991+1655A>G) in the *CEP290* gene, underlying recessive Leber congenital amaurosis (LCA; MIM:611755). This mutation results in the generation of a cryptic splice donor site leading to a 128-nt pseudoexon with a premature stop codon between exons 26 and 27. AONs used to block the pseudoexon showed successful restoration of the original mRNA both in vivo and in vitro [22,24,25,26,27] and have recently shown promising results in the first clinical trial with AONs for IRDs [32].

Another mutation that causes a pre-mRNA splicing defect and is amenable to AON therapy is the c.4539+2001G>A variant in *ABCA4* [13,14,15], which is recurrently found in the Belgian and Dutch STGD1 population. Recently, our group described the molecular mechanism by which c.4539+2001G>A and the adjacent c.4539+2028C>T mutations in *ABCA4* lead to insertion of a retina-specific 345-nt pseudoexon that is predicted to result in premature termination of protein synthesis (p.Arg1514Leufs*36). The c.4539+2001G>A variant enhances a predicted exonic splice enhancer and creates a new SRp55 motif. This was the first reported insertion of a pseudoexon into a retinal gene due to the creation of new exonic splicing enhancer (ESE) motifs rather than the generation of new cryptic splice sites, although other examples have been described previously [33,34]. By using AON technology, we were able to restore correct splicing with two of the four AONs (AON1–4) that were used (AON1 and AON4) [18].

In this study, we performed an in-depth screening of a large set of AONs targeting the entire pseudoexon region to identify the most effective AON(s) against the splicing defect caused by the c.4539+2001G>A mutation. In total, 26 AONs were screened in retinal precursor cells differentiated from patient-derived induced pluripotent stem cell (iPSC), and their efficacy in correcting splicing defects was assessed. Subsequently, properties of the most effective AONs were compared in order to identify potential parameters for a better design of AONs in the future. 

## 2. Materials and Methods 

### 2.1. Study Design

The objectives of this study were to (1) perform an in-depth screening of AONs targeting the pseudoexon introduced by the recurrent c.4539+2001G>A deep-intronic variant in *ABCA4*, (2) identify the best AON(s) to correct the pre-mRNA splicing defect caused by this mutation using patient-derived photoreceptor precursor cells (PPCs), and (3) identify potential correlations between AON characteristics and their efficacy that can provide new insights into a better AON design. Twenty-two new AONs targeting the pseudoexon were designed and tested together with four previously described AONs [18]. Fibroblast cells obtained from a skin biopsy of a Stargardt individual carrying the deep-intronic variant were cultured, reprogrammed into iPSCs, and subsequently differentiated to PPCs. All 26 AONs and two sense oligonucleotides (SONs) were designed along the pseudoexon. Upon AON delivery, subsequent RNA analysis by RT-PCR was performed to assess the efficacy of the splicing redirection for each AON. After semiquantification of the rescue, AONs were classified into different groups, and the properties of the AONs were compared to identify parameters that could improve the AON design. Two separate differentiation experiments were performed. RNA analysis was performed in triplicate to reduce technical variability.

### 2.2. AON Design

Previously, four AONs were designed targeting the top SC35 motifs and the mutation itself [18]. For the detailed screening that was the subject of this study, the entire pseudoexon plus the flanking regions were analyzed for their RNA structure to identify the open and closed regions. Subsequently, AONs were designed according to previously described guidelines independently of the potential motifs that they were targeting [35,36]. All AON sequences and properties are provided in Table 1. After AON design, targeted regions were analyzed to predict potential exonic splicing enhancer (ESE) motifs using either an ESE finder (http://krainer01.cshl.edu/cgi-bin/tools/ESE3/esefinder.cgi?process=home), which allows for the detection of SRSF1, SRSF2, SRSF5, and SRSF6, or using RBPmap (http://rbpmap.technion.ac.il/index.html), which allows for the identification of 94 potential binding sites for RNA binding proteins. All AONs were 2’*O*Me-PS (2’*O*-methyl phosphorothioate) and were purchased from Eurogentec (Liege, Belgium). Sequences and general parameters of the 26 AONs and 2 SONs are depicted in Table 1. 

### 2.3. Subjects

A skin biopsy was collected from a Dutch individual with STGD1 carrying the *ABCA4* variants c.4539+2001G>A (p.Arg1514Leufs^∗^36) and c.4892T>C (p.Leu1631Pro) to establish a fibroblast cell line, as described previously [18]. Our research was conducted according to the tenets of the Declaration of Helsinki and after gathering written informed consent from the STGD1 individual. The procedures for obtaining human skin biopsies to establish primary fibroblast cell lines were approved by the local ethical committee (2015-1543).

### 2.4. iPSC Differentiation into Photoreceptor Precursor Cells (PPCs)

Fibroblast cells were reprogrammed into iPSCs, as previously described [18]. PPCs were obtained after following a 2D differentiation protocol [37]. Briefly, iPSCs were dissociated with ReLeSR (Stemcell Technologies) and plated in 12-well plates coated with matrigel (Corning, Tewksbury, MA, USA) to form a monolayer. Essential-Flex E8 medium was changed to differentiation medium (CI) when reaching confluence. The CI medium consisted of DMEM/F12 supplemented with nonessential amino acids (NEAA, Sigma Aldrich, Saint Louis, CA, USA), B27 supplements (Thermo Fisher Scientific, Waltham, MA, USA), N2 supplements (Thermo Fisher Scientific), 100 ng/µL of insulin growth factor-1 (IGF-1, Sigma Aldrich), 10 ng/µL of recombinant fibroblast growth factor basic (bFGF, Sigma Aldrich), 10 µg/µL of Heparin (Sigma Aldrich), 200 µg/mL of recombinant human COCO (R&D Systems, Minneapolis, MN, USA), and 100 µg/mL of Primocin (Invivogen, Toulouse, France). Half of the medium was replaced every day for 30 days. On day 28, PPCs were treated with 1 µM of AON. AONs were first mixed with the medium without any transfection reagent and were subsequently added to the cells. Twenty-four hours later, cycloheximide (CHX) was added to the medium (final concentration 100 µg/mL), and on day 30 (48 h post-AON delivery and 24 h post-CHX treatment), cells were collected.

### 2.5. RNA Analysis

RNA was isolated from patient-derived PPCs using the Nucleospin RNA kit (Machery Nagel, Düren, Germany) following the manufacturer’s instructions. One microgram of total RNA was used for cDNA synthesis using SuperScript VILO Master Mix (Thermo Fisher Scientific) and was subsequently diluted with H_2_O to a final concentration of 20 ng/µL. Reverse transcription-PCR (RT-PCR) was performed with 10 µM of each primer, 2 µM of dNTPs, 2.5 mM of MgCl_2_, 1 U of Taq polymerase (Roche, Basel, Switzerland), and 80 ng of cDNA in a total reaction of 25 µL using the following PCR conditions: 2 min at 94 °C, followed by 35 cycles of 30 s at 94 °C, 30 s at 58 °C, and 70 s at 72 °C, with a final extension of 2 min at 72 °C. Actin was amplified to serve as a loading control. All PCR products were resolved on 2% agarose gels and were confirmed by Sanger. Fiji software was used to perform a semiquantitative analysis of the bands in which the values were normalized against the housekeeping gene *ACTB* [38]. For that, the band representing the 345-nt pseudoexon, plus half of the value of the heteroduplexes, and the partial pseudoexon skipping band were counted as aberrant. The other half of the heteroduplexes together with the correct band were considered to be correct transcripts. We observed a nonspecific band that was not considered for the analysis. The list of primers is provided in the Supplementary Materials, Table S1. 

### 2.6. qPCR

The cDNA samples were obtained as described above from iPSCs at day 0, and the nontreated PPCs at day 30 (both replicates) were used for quantitative real-time PCR (qPCR) to assess the differentiation process: qPCR was performed using GoTaq qPCR master mix (Promega, Madison, WI, USA). Three technical replicates were done for each of the two biological replicates. The list of primers is provided in the Supplementary Materials, Table S1. 

### 2.7. AON Classification and Common Properties

Once the rescue was assessed, AONs were classified into 5 groups: Highly effective (>75% correction), effective (between 75% and 50%), moderately effective (between 50% and 25%), poorly effective (between 25% and 0%), and noneffective (no correction detected). For the study of the properties of each group, the groups poorly effective and noneffective were combined into one single group, as well as the highly effective and effective groups, generating three new groups: Effective, moderately effective, and poorly effective. Using this information, several potential correlations between AON properties, target motifs, and their efficacy were assessed, with the aim of establishing possible improvements in the AON design. Statistical analyses were performed using GraphPad Prism. Given the low numbers for some of the groups, normality could not be assessed, and therefore nonparametric tests were used. 

## 3. Results

Previously, we showed that the variants c.4539+2001G>A and c.4539+2028C>T cause the insertion of a 345-nt pseudoexon in a retina-specific manner. Four AONs (AON1 to AON4) targeting this region were designed according to previously described guidelines [35,36,39] and were assessed in PPCs. Our results showed that two AONs (AON1 and AON4) were able to restore correct *ABCA4* splicing by skipping the pseudoexon in a mutation-dependent manner. AON1 was specific for the c.4539+2001G>A variant and was not able to correct the splicing defect caused by the c.4539+2028C>T mutation, suggesting that one nucleotide mismatch can already impair rescue efficacy. Here, we screened the entire pseudoexon region in order to identify potential new targets that can promote splicing redirection with a higher efficacy by designing 22 new AONs.

### 3.1. Screening and Selection of AONs

**Figure 1:**
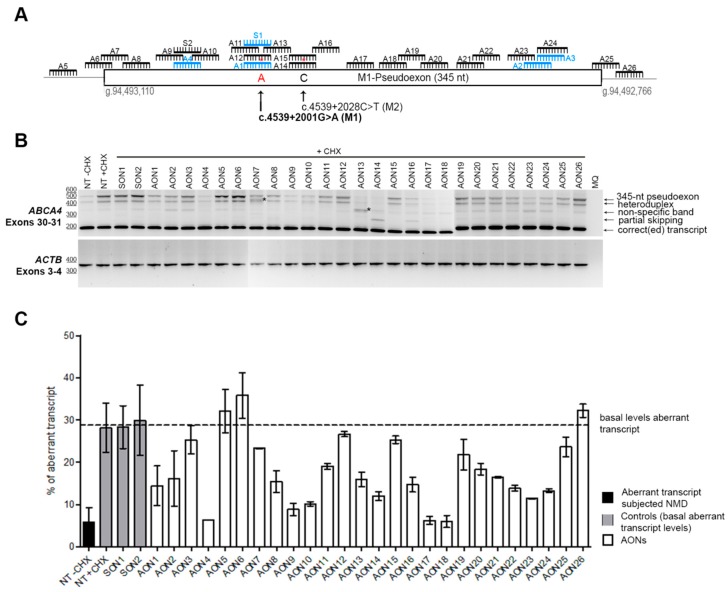
AON-based pseudoexon skipping efficacy. (**A**) Schematic representation of the 345-nt pseudoexon insertion caused by the c.4539+2001G>A mutation and the location of the 26 AONs and the 2 sense oligonucleotides (SONs). Blue oligonucleotides refer to previously studied molecules [18]. Red asterisks represent mismatches with the pseudoexon sequence created only by the c.4539+2001G>A mutation (namely an A at position c.4539+2001 and a C at position c.4539+2028). (**B**) Representative image of an RT-PCR performed on patient-derived photoreceptor precursor cells (PPCs) upon AON treatment. *ACTB* was used to normalize samples. An heteroduplex band was observed in all samples, containing the correct and the pseudoexon-included transcripts. AON-derived partial skipping was observed in samples AON14 and AON16. Double bands highlighted with an * (lanes AON7 and AON13) indicate artifacts derived from the AON treatment, and splice sites could not be identified upon Sanger sequencing. In most of the lanes, we identified a PCR artifact (nonspecific band). (**C**) Percentage of aberrant transcript after semiquantification. NT: Nontreated; CHX: Cycloheximide. Error bars indicate average ± SD.

The entire pseudoexon (345 nt) together with its flanking regions were subjected to AON design. A total of 22 new AONs were designed throughout the entire region (Figure 1A). The AON design parameters, such as melting temperature (Tm), GC content, and free energy were assessed in order to have optimal sequences when possible (e.g., Tm > 48 °C, GC content between 40% and 60%). Subsequently, to further assess what AONs were targeting, we predicted the RNA structure of the region using mfold software [40]. We also checked the ESE motifs that were present in the region using an ESE finder (http://rulai.cshl.edu/) or the potential RNA binding protein sites using RBPmap (http://rbpmap.technion.ac.il/). Overall, all AONs were covering the pseudoexon or its splice sites and were targeting all types of regions (predicted to be more closed or open) and motifs. 

**Figure 2:**
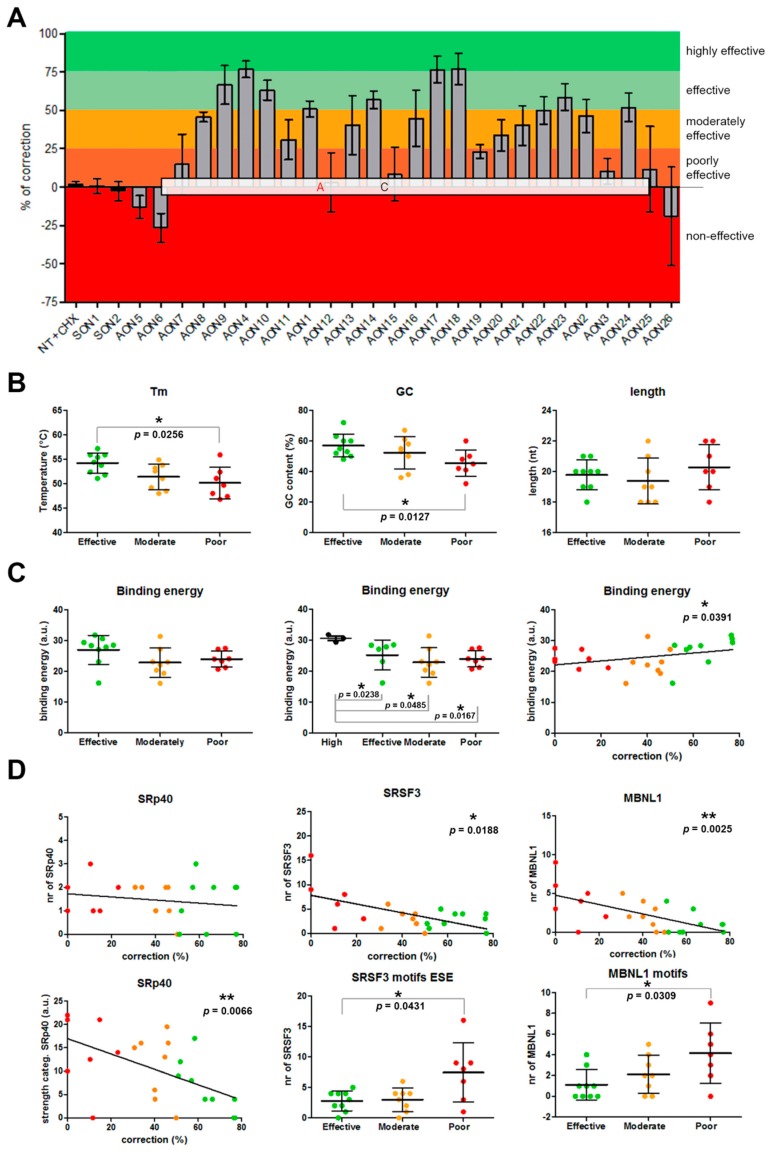
Assessment of AON efficacy and correlations. (**A**) Percentage of correction for each AON. AONs are located according to their position in the c.4539+2001G>A-specific pseudoexon. Colors indicate the efficacy classification that was established. (**B**–**C**) Representation of the statistical analyses for general parameters taken into account for AON design. (**D**) Analysis of the influence of certain motifs in AON efficacy. Error bars in all graphs indicate average ± SD.

Patient-derived iPSCs heterozygously carrying the c.4539+2001G>A mutation in conjunction with another *ABCA4* mutation on the other allele were differentiated into PPCs. Differentiation of the cells was assessed by qPCR. The results showed a differentiation toward retinal lineage with a clear increase in *ABCA4* expression (Supplementary Materials, Figure S1). As we already described, the 345-nt pseudoexon was only visible upon inhibition of nonsense-mediated decay (NMD) (Figure 1B). Therefore, PPCs were first treated with the corresponding AON and after 24 h were subjected to cycloheximide (CHX) treatment to inhibit NMD. RNA analysis was performed by RT-PCR (Figure 1B). We then semiquantified the amount of aberrant transcript (Figure 1C). Remarkably, three AONs were able to almost completely rescue the splicing defect (AON4, AON17, and AON18). Interestingly, we also observed that four AONs (AON7, AON13, AON14, and AON16) caused the appearance of additional bands. Some of them represented partial pseudoexon skipping, while others turned out to be potential artifacts due to mis-splicing, although we could not exactly determine the splicing sites. Sequencing results determined that the partial-exon skipping observed in AON14 and AON16-treated samples was a partial skipping of exon 30 (previously described in Reference [18]) together with partial skipping of the first 142 nt of the pseudoexon (splice acceptor site in c.4539+2035). In the case of AON13, we identified partial pseudoexon exclusion, but we could not determine the splice acceptor site. In the case of AON7, we could not determine both splice sites (acceptor and donor), and it was probably an aberrant mRNA caused by the AON treatment. Using the average of the cells not treated with AONs but subjected to CHX treatment and the ones treated with the sense oligonucleotide (SON), we established the basal levels (~29%) of the *ABCA4* aberrant transcript (Figure 1C). These values were used to establish the percentage of correction for each AON (Figure 2A). Five groups were determined: Highly effective (correction >75%, *n* = 3), effective (75%–50%, *n* = 6), moderately effective (50%–25%, *n* = 8), poorly effective (25%–0%, *n* = 9), and noneffective (0% or even increasing the amount of pseudoexon). These groups are depicted in Figure 2A according to different colors in the graph.

### 3.2. Analysis of the Properties of the AONs 

Once the efficacy of all AONs was estimated, we subdivided them into three larger groups: Effective (*n* = 9 comprising all AONs with an efficacy > 50%), moderately effective (*n* = 8 with efficacies between 25% and 50%), and poorly effective (*n* = 7, the rest). AONs containing a mismatch (AON12 and AON15) were not used in these analyses, as the decrease in the efficacy was due to the mismatch. This was shown by the fact that both AON1 and AON14 (perfectly matching the c.4539+2001G>A allele, but containing a mismatch for the PE induced by the c.4539+2028C>T mutation) correct the splice defect associated with c.4539+2001G>A.

We first analyzed the basic parameters: Melting temperature (Tm), GC content, and length (Figure 2B). We found a statistically significant correlation (two-tailed Spearman test) for the Tm and GC content parameters (*p* = 0.0012 and *p* = 0.0041, respectively). In both cases, the higher the value was, the more efficient the AON was (Supplementary Materials, Figure S2). The Tm average of the three groups ranged from 54.21 °C (effective) to 50.19 °C (poorly effective). The differences in Tm between groups was statistically different (*p* = 0.0292, nonparametric one-way ANOVA), while the GC content was nearly significant (*p* = 0.0611). When comparing all different groups separately, statistically significant differences were observed between the groups of effective AONs and poorly effective AONs for Tm (*p* = 0.0256, Mann–Whitney test) and GC content (*p* = 0.0127). No differences were observed for the length of the AONs.

Other important parameters when designing AONs are the free energy of the AON molecule itself, the dimer, and the binding energy to the target. The free energy of the AON molecule and its dimer did not show any correlation nor difference between groups. Interestingly, the binding energy showed a significant correlation (*p* = 0.0391) with efficacy. We analyzed the groups separately, and although no statistically significant differences were found, the effective group showed a trend toward significance when compared to the moderately effective (*p* = 0.0673, Mann–Whitney test) and the poorly effective (*p* = 0.0712, Mann–Whitney test) groups. Consistently, the highest binding energies corresponded to the three most effective AONs (AON4, AON17, and AON18). Interestingly, when these three values were separated, the tendency of the effective group disappeared. In contrast, these three AONs only showed a statistically significant higher binding energy compared to the other three groups (Figure 2C). 

Next, we checked the common serine and arginine-rich splicing factors (SRSFs): SF2 (SRSF1), SC35 (SRSF2), SRp40 (SRSF4), and SRp55 (SRSF5)) using an ESE finder (Supplementary Materials, Figure S3). All motifs that were partially or completely covered by an AON were counted and assigned to the particular AON. We did not find any correlation between the percentage of correction and the presence of these motifs. However, we noticed that the strength of some of the motifs was different between groups. For that, we categorized all the motifs found by assigning them a number according to the strength of the predicted score (lowest = 1, second lowest = 2, etc.). After that, the average for each AON was calculated. Interestingly, a statistically significant correlation (*p* = 0.0066, two-tailed Spearman test) was observed for the categorized SRp40 motifs. In this case, the strongest motifs were correlating with the poorly or moderately effective AONs, and the lowest with the effective ones (Figure 2D). However, when pooling all groups together, differences were not statistically significant. No other differences were detected for any of the other three SRSF motifs, except for SC35, where significant differences were observed when comparing effective versus moderately effective AONs (*p* = 0.0316, Mann–Whitney test; Supplementary Materials, Figure S3). 

Finally, we used RBPmap to predict potential RNA protein-binding motifs. Again, each motif detected in the region was assigned to each AON when partial or complete overlap occurred. First, manual filtering of the motifs was done by checking which motifs were common to all AONs in each group. Unfortunately, none of the motifs were shared between all effective AONs. AON18 was the one behaving differently than the rest, almost not sharing any motif with the others. When AON18 was left out of the filtering, SRSF3 appeared as a common motif not only in the effective group, but also in the poorly and moderately effective groups. When assessed in more detail (Figure 2D), a statistically significant negative correlation was observed (*p* = 0.0188, two-tailed Spearman test). The analysis per group revealed a significant difference between the effective and poorly effective groups (*p* = 0.0431) and close to significance between the moderately and poorly effective groups (*p* = 0.0622). Categorized SRSF3 did not show significant differences. The second most recurrent motif in the poorly effective group was MBNL1. Given the fact that all but one poorly acting AON contained this predicted motif, we performed statistical analyses to determine whether the presence of MBNL1 motifs correlated with the lower performance of some AONs. Indeed, a significant correlation was observed (*p* = 0.0025), implying an association between the presence of these motifs and a low AON performance (Figure 2D). When differences between groups were assessed, only the effective group showed a statistically significant lower number of MBNL1 when compared to the poorly effective group (*p* = 0.0309). The total amount of motifs detected by RBPmap showed a nearly significant negative correlation with efficiency (*p* = 0.0522). However, when groups were analyzed separately, this trend completely disappeared (Supplementary Materials, Figure S4).

## 4. Discussion

The fact that the eye is an isolated, immunoprivileged, and easily accessible organ makes it a very attractive model for molecular therapies. In addition, IRDs are progressive diseases that offer a window of opportunity for treatment. However, the high genetic heterogeneity in IRDs hampers the development of new therapies. AONs have been shown to be an auspicious approach to treat splicing defects causing IRDs. They have been shown to be safe and easy to deliver to the retina without the necessity of any vector. Furthermore, recent results from a phase 1/2 clinical trial to correct the splicing defect introduced by a deep-intronic mutation in *CEP290* showed the great potential that these molecules hold. In this study, we screened 26 AONs targeting a pseudoexon product of a recurrent deep-intronic mutation (c.4539+2001G>A) in the *ABCA4* gene with the intention of identifying the most promising AON molecules to eventually treat STGD1.

We designed and tested 26 different AONs located across the 345-nt pseudoexon. In total, three highly effective AONs were identified: AON4, AON17, and AON18. All of them showed similar efficacies (76.42%–76.97%). Another six AONs (AON1, AON9, AON10, AON14, AON23, and AON24) corrected the pre-mRNA splicing defect with an efficacy of more than 50%. Previously, we showed that AON1 was only effective when the change c.4539+2001G>A was present, as the pseudoexon containing the change c.4539+2028C>T was not removed. This highlighted the fact that for 2’*O*Me/PS chemically modified AONs, one mismatch could dramatically affect the splicing redirection efficacy. Here, we demonstrated again that one mismatch was enough to prevent AON-based splicing modulation. For that, we generated the same AON1 but with a wild-type nucleotide (AON12). Furthermore, we also designed an AON specific for the c.4539+2028C>T variant (AON15) and a corresponding AON with a wild-type change (AON14). As expected, the mutation-specific AON (AON15) did not redirect splicing in this cell line, while the one perfectly matching the target did (AON14). Interestingly, AON14, although its correction efficacy was around 50%, is not considered a very promising AON for future studies since it was one of the four AONs (together with AON7, AON13, and AON16) that showed novel aberrant bands in RT-PCR. In this particular case, both AON14 and AON16 caused an unexpected (AONs binding on top of the novel splice acceptor site at position c.4539+2035) partial pseudoexon exclusion together with an already described partial exon 30 skipping [18]. However, this was clearly induced by these two AONs, which bind in the same region. However, for AON7 and AON13, we were not able to determine how the aberrant band was generated due to the lack of predicted splicing sites, and therefore we considered them artifacts. We previously observed a similar effect for another variant, and we concluded that this was either a PCR artifact or, due to the fact that AONs can interfere with RNA structure and the splicing machinery, some aberrant mis-spliced transcripts that may have appeared [20].

After classifying the AONs into three groups, we tried to identify common properties that could eventually lead to a better AON design. When analyzing the groups, we found correlations with the Tm and the GC content. Previously published guidelines [35,36,39] have indicated that Tm should be above 48 °C and the GC content between 40% and 60%. Based on our analyses, it seems that if the temperature is higher than 51 °C and the GC content close to 60%, the chances of designing an AON with a good efficacy are higher. According to previous guidelines, the binding energy should stay between 21 and 28. However, our best AON molecules had binding energies to the target of more than 30. Moreover, based on correlations that we were able to identify, AONs targeting predicted SRSF3 or MBNL1, as well as strong predicted SRp40 motifs, might show poorer efficacies. Although some of these parameters have been shown before to be relevant to AON design, it is important to mention that the efficacy of an AON molecule also can depend on the type of cell, tissue, or organ. In that sense, the parameters established above might be valid only for AONs delivered to retinal cells, and further confirmation in cells of other origins needs to be addressed. Unfortunately, this comparison was not possible in our case due to the fact that although *ABCA4* is lowly expressed in fibroblast cells, the splicing defects observed for some mutations, including c.4539+2001G>A, are not recapitulated in those cells. Most probably this is because the splicing retinal machinery may have different efficacies or recognition sites [41,42]. 

In our previous studies, AON1 showed an efficacy of ~75% [18]. However, in this study, only ~51% correction was detected, although the PPCs were derived from the same patient iPSC line. One explanation could be that in our previous study, we only detected around 25% of pseudoexon insertion, while in this study we were able to detect more pseudoexon-including transcripts (~30%). This could therefore have modified the correction ratio. In addition, the inhibition of NMD by CHX is not always complete, and therefore variability in the detection of the pseudoexon transcript (subjected to NMD) might have been variable between experiments and samples. Another possible explanation could be differences between the AON batches. All of these factors, either alone or in combination, could have influenced the differences observed between the two studies. 

Finally, as discussed above, AON4, AON17, and AON18 showed the highest efficacies. However, AON18 did not show that much similarity to the other two AONs other than a high binding energy. AON18 did not share obvious targeted predicted motifs with either of the two highly effective AONs (Supplementary Materials, Figure S5). We also checked the secondary structure of the RNA and all three AON target partially closed regions. Therefore, the slightly higher efficacy might have been related to the binding to the target itself and the disruption of the secondary structure rather than the motifs that were blocked by the molecule. In addition, when comparing the sequences to see which one could be a potential candidate for further development, we observed that all three AONs contained stretches of Gs and Cs (which are recommended to be avoided). AON4 and AON17 contained a G stretch of four and three Gs, respectively, while AON18 contained two stretches of three and four Cs. Nevertheless, given their efficacy, all three molecules might be potential good candidates for further therapeutic studies. 

## 5. Conclusions

In conclusion, we designed 26 AONs targeting a 345-nt pseudoexon caused by the recurrent c.4539+2001G>A deep-intronic mutation in *ABCA4*. In total, nine AONs showed promising efficacies (correction above 50%). We identified three AONs promoting a correction superior to 75%. For AON design, we suggest increasing the minimum Tm to 50 or 51 °C, the GC content close to 60%, and the binding energy to around 30 to target retinal pseudoexons, although this needs to be tested and confirmed using other targets. Overall, we demonstrated that AON-based splicing modulation holds great potential for treating Stargardt disease caused by splicing defects in *ABCA4*. 

## 6. Patents

A.G. and R.W.J.C. are inventors on a filed patent (PCT/EP2017/1082627) that is related to the contents of this manuscript.

## Figures and Tables

**Table 1:** Antisense oligonucleotide (AON) sequences and general parameters.

AON#	Sequence (5′to 3′)	L	Tm	GC	FE-A	FE-D	BE	Remarks
AON1	ACAGGAGUCCUCAGCAUUG	19	51.1	53	−0.1	−12.4	16.2	Specific for c.4539+2001G>A-pseudoexon Previously reported in Reference [18]
AON2	UUUUGUCCAGGGACCAAGG	19	51.1	53	−1.6	−15.6	23.1	Previously reported in Reference [18]
AON3	CUGUUACAUUUUGUCCAGG	19	46.8	42	−0.9	−7.3	20.7	Previously reported in Reference [18]
AON4	GGGGCACAGAGGACUGAGA	19	55.4	63	−0.8	−5.9	30.6	Previously reported in Reference [18]
AON5	GAGAGAAAAUAUUGCUUGAGAA	22	47.4	32	1.7	−5.0	27.5	
AON6	GCAGAUGAGCUGUGAUUCAA	20	49.7	45	−2.5	−8.8	24.0	
AON7	UAUGAUGCAGCAGAUGAGCUG	21	52.4	48	−3.9	−12.2	24.1	
AON8	UGGGAUCCCUAUGAUGCAGC	20	53.8	55	−1.1	−17.4	19.4	
AON9	AGAGGACUGAGACAAGUUCC	20	51.8	50	−4.2	−10.0	23.1	
AON10	GCUUCCUCUUGGGGCACAGA	20	55.9	60	−5.1	−12.0	28.4	
AON11	CCUCAGCAUUGACAGCAA	18	48	50	−0.6	−3.2	16.1	
AON12	ACAGGAGCCCUCAGCAUUG	19	53.2	58	−0.4	−9.3	11.1	One mismatch in c.4539+2001G>A-pseudoexon
AON13	UGGAGGCAGCCACAGGAG	18	54.9	67	−1.3	−11.8	31.4	
AON14	GAUGCUGGAGGGUUUUGAGUG	21	54.4	52	−1.7	−12.6	27.1	One mismatch in c.4539+2028C>T-pseudoexon Perfect match in c.4539+2001G>A-pseudoexon
AON15	GAUGCUGGAGAGUUUUGAGUG	21	52.4	48	−1.7	−14.2	20.2	Specific for c.4539+2028C>T-pseudoexon One mismatch in c.4539+2001G>A-pseudoexon
AON16	GCCUUGACGUCCUGAUGCU	19	53.2	58	1.4	−10.3	20.4	
AON17	GCCAAGAGCUCAGGGUACAG	20	55.9	60	−0.9	−19.9	31.8	
AON18	CUUGGCCUCCCCUCCCUC	18	57.2	72	1.4	−8.3	29.4	
AON19	AACACCAUGUAGGUAGGC	18	48	50	−1.6	−6.8	21.2	
AON20	GUUUAGGAAAUGAAACACCAUG	22	49.2	36	−0.7	−4.5	23.0	
AON21	GACCGCGUGGAAGUAAGG	18	52.6	61	−0.3	−14.9	22.1	
AON22	AUAAGUUUCUAAGCUGGACAG	21	48.5	38	−0.4	−8.1	27.2	
AON23	GGACCAAGGACCAACACUAC	20	53.8	55	−0.6	−9.7	27.9	
AON24	GGCUGUUACAUUUUGUCCAGG	21	52.4	48	−1.0	−7.5	28.5	
AON25	GGCAGGAACUGGCUUGCCUU	20	55.9	60	−8.6	−20.2	27.2	
AON26	AGAAGUGAAAGAAAAUGGCAGG	22	51.1	41	1.9	−3.0	23.3	
SON1	CAAUGCUGAGGACUCCUGU	19	51.1	53	−0.7	−11	6.0	Sense sequence of AON1 Previously reported in Reference [18]
SON2	UCUCAGUCCUCUGUGCCCC	19	55.4	63	−0.9	−5.6	3.4	Sense sequence of AON4

The nucleotides underlined represent the possible mismatch in relation to the mutation present in the pseudoexon (c.4539+2001G>A or c.4539+2028C>T). L: Length in nt; Tm: Melting temperature in °C; GC: GC content in %; FE-A: Free energy AON molecule; FE-D: Free energy AON dimer; BE: Binding energy to the target region. All energy values are in arbitrary units obtained using RNAstructure software (https://rna.urmc.rochester.edu/RNAstructureWeb/Servers/bifold/bifold.html).

## Supplementary Materials

The following are available online at https://www.mdpi.com/2073-4425/10/6/452/s1, Table S1: List of primers; Figure S1: Analysis by qPCR of iPSCs and photoreceptor precursor cells; Figure S2: Representation of the correlation on general parameters; Figure S3: Representation of the analyses performed on regular exonic splicing enhancers; Figure S4: Analysis of all predicted RNA binding sites using RBPmap; Figure S5: Schematic representation of the percentage of correction with exonic splicing enhancers and silencers.
