# Supplementary material for: Antisense Oligonucleotide Screening to Optimize the Rescue of the Splicing Defect Caused by the Recurrent Deep-Intronic ABCA4 Variant c.4539+2001G>A in Stargardt Disease
Source: Genes (Basel). 2019 Jun 14;10(6):452. doi: 10.3390/genes10060452 (PMC6628380; doi:10.3390/genes10060452)
Supplement: Supplementary file 1 [file genes-10-00452-s001.pdf]

Supplementary Material

Supplementary Table S1- List of primers

List of primers		
Gene	**Forward primer sequence 5′ → 3′**	**Reverse primer sequence 5′ → 3′**
**RT-PCR**		
ABCA4	AAACATCACCCAGCTGTTCC	GAAGTCGGAGATGTTCCTGTC
ACTB	ACTGGGACGACATGGAGAAG	TCTCAGCTGTGGTGGTGAAG
**qPCR**		
PAX6	CCGGCAGAAGATTGTAGAGC	GCCCGTTCAACATCCTTAGT
CRX	GCCCCACTATTCTGTCAACG	CTTCAGAGCCACCTCCTCA
RCV1	ACACCAAGTTCTCGGAGGAG	ACTTGGCGTAGATGCTCTGG
OPN1SW	ACCATTGGTATTGGCGTCTC	GGAGAGAGGCACAATGAAGC
OPN1M/LW	GTGGTCACTGCATCCGTCTT	ACGGTCTCTGCTAGGTCAGC
PDE6C	TTGGGAACAAGGAGATCTGG	GGCTCCTCCTTCTTGCTTTT
PDE6H	TACTCTGCCTGCTCCAGCTT	GAGAGTGGCAGAACCTCTGG
RHO	TCATCATGGTCATCGCTTTC	CATGAAGATGGGACCGAAGT
NRL	GGCTCCACACCTTACAGCTC	AGCCAGTACAGCTCCTCCAG
ABCA4	CATCCTGTTCCACCACCTCA	CTGTGTCCTCCAACATGGCT
OTX2	TATCTTAAGCAACCGCCTTACG	GGAGGGGTGCAGCAAGTC
RPE65	GCCCTCCTGCACAAGTTTGACTTT	AGTTGGTCTCTGTGCAAGCGTAGT
OCT3/4	GTTCTTCATTCACTAAGGAAGG	CAAGAGCATCATTGAACTTCAC
VMD2	TAGAACCATCAGCGCCGTC	TGAGTGTAGTGTGTATGTTGG
NR2E3	CATCTACAGGTGCCAGGTG	CCTGCAGGCACTTCTTCA

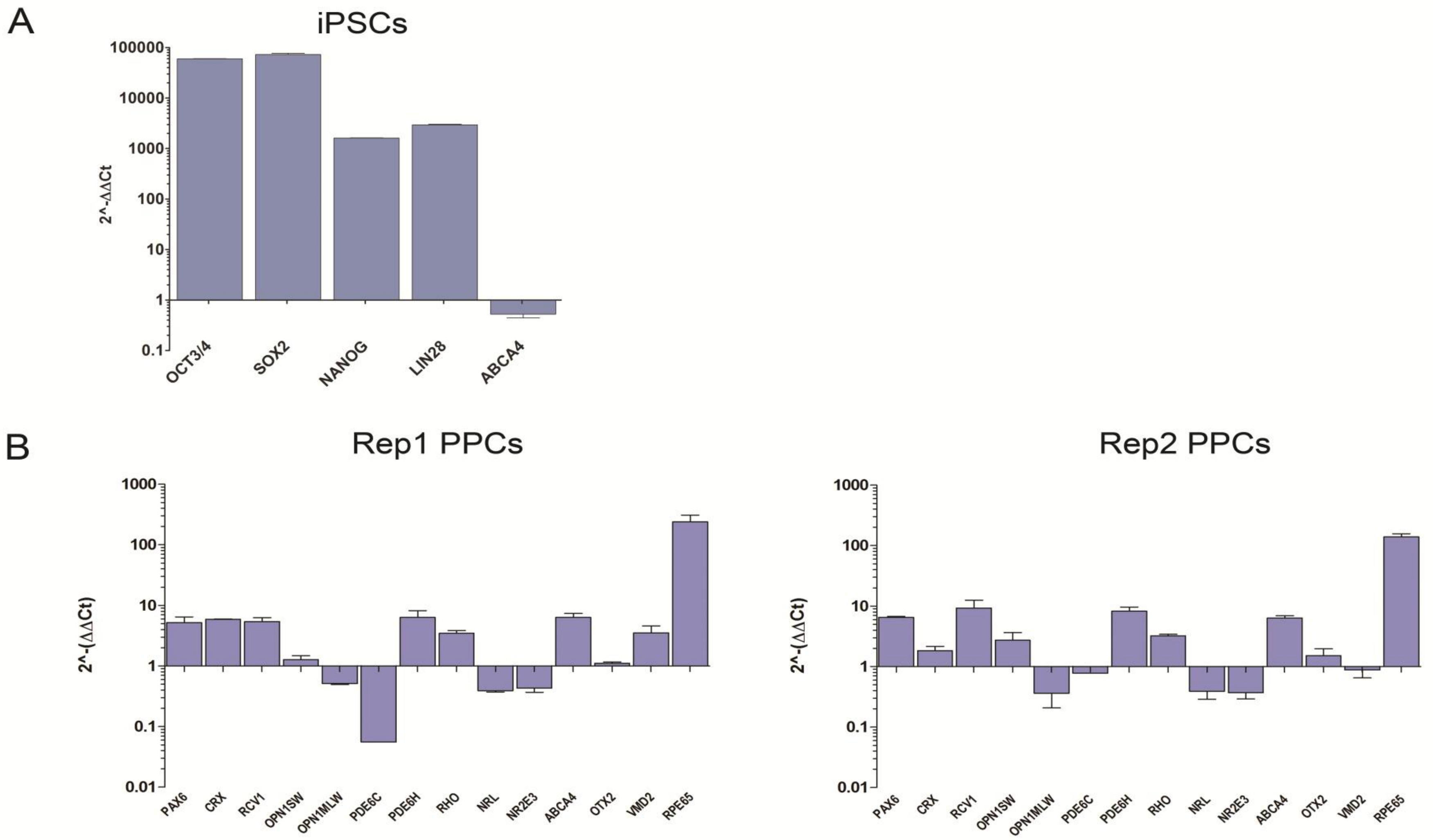

Figure S1 – Analysis by qPCR of (A) iPSCs and (B) photoreceptor precursor cells (PPCs). Analysis of the pluripotency and retinal precursor markers. PPC: Photoreceptor precursor cells; Rep1 and Rep2: Replicate 1 or replicate 2. In all cases there was a significant increase of *ABCA4*.

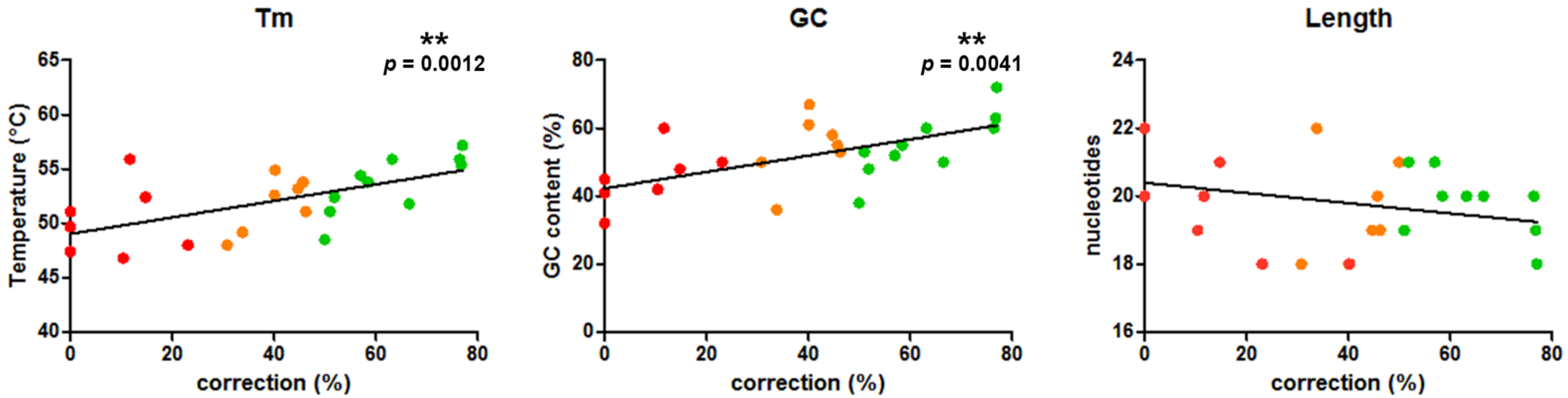

Figure S2 – Representation of the correlation on general parameters

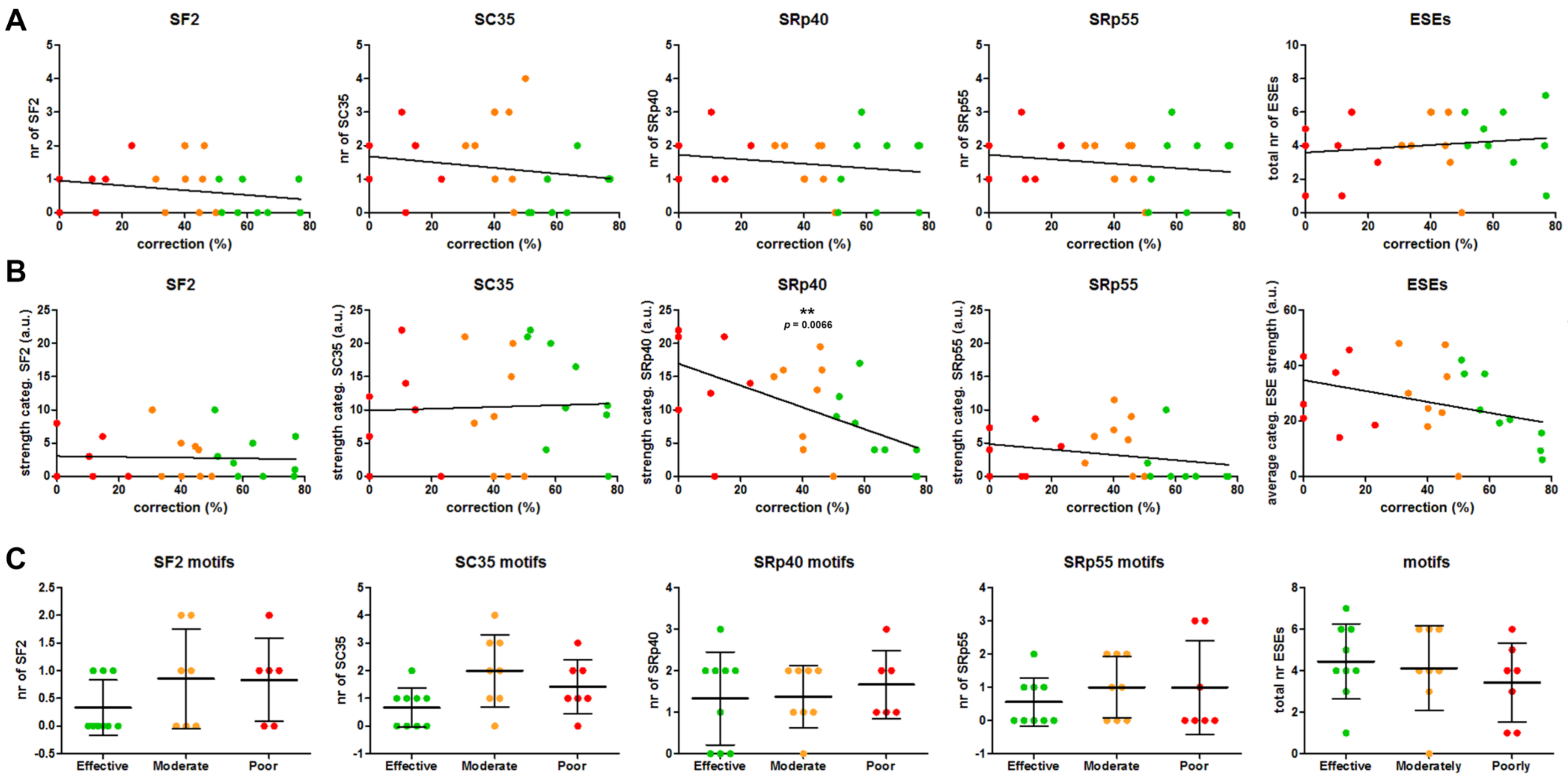

Figure S3 – Representation of the analyses performed on regular exonic splicing enhancers (ESEs). (A) Correlation based on the correction efficacy and the number of motifs for each ESE or all together (ESEs graph). **(B)** Correlation between efficacy and strength of the ESEs. **(C)** Analysis per groups according to the strength of the motifs.

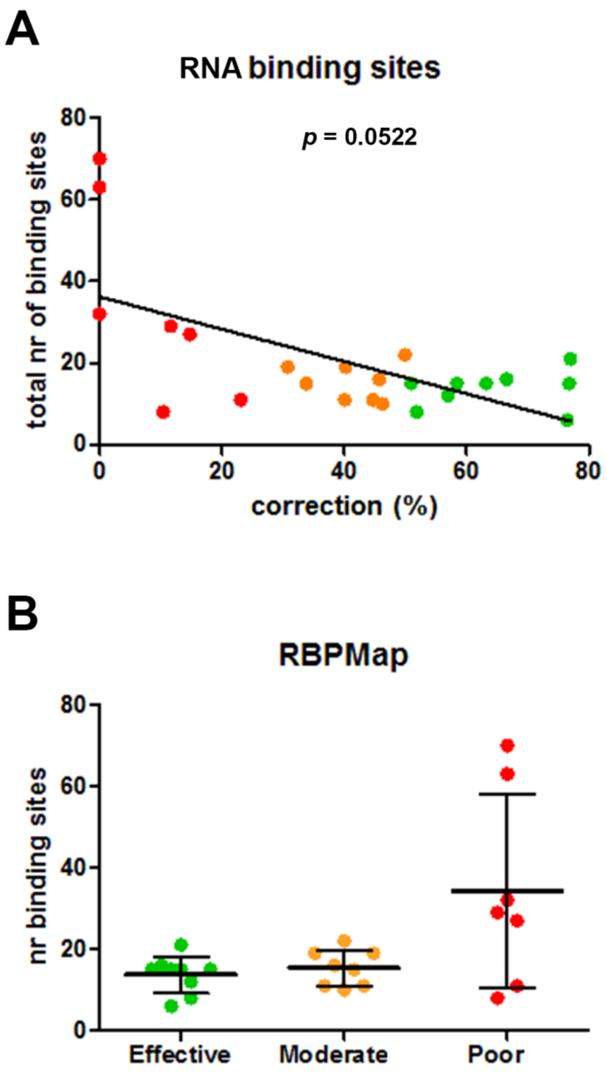

Figure S4 – Analysis of all predicted RNA binding sites using RBPmap. (A) correlation between efficacy and binding sites. (B) Analysis per groups.

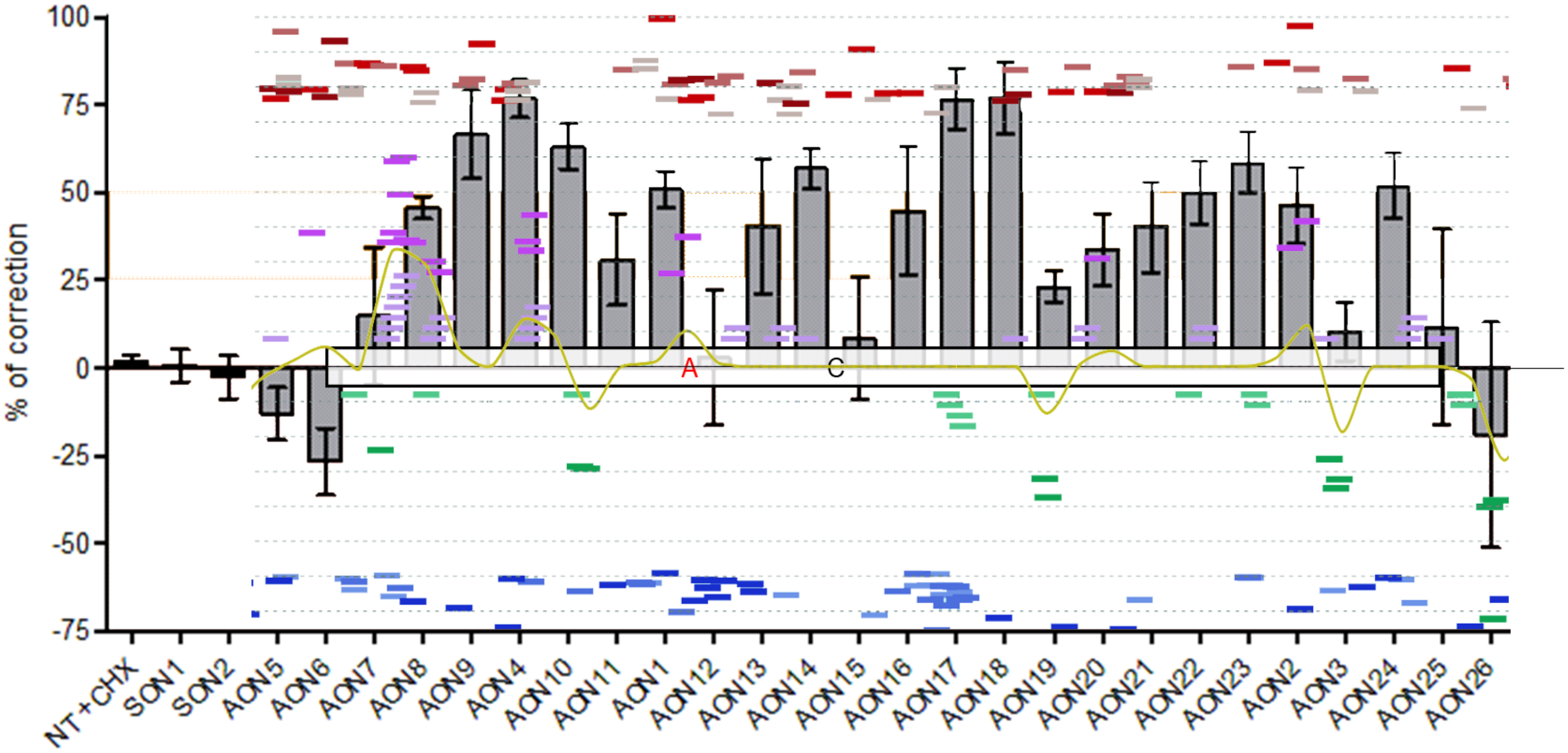

Figure S5 – Schematic representation of the percentage of correction with exonic splicing enhancers and silencers. Enhancers (ESEs, red and pink boxes in the positive part of the Y axis) and silencers (ESSs, green and blue boxes in the negative part of the Y axis) identified using Human Splicing Finder software are depicted in the figure. Yellow line represents the ESEs/ESSs relative strength.
